# Applying the Properties of Neurons in Machine Learning: A Brain-like Neural Model with Interactive Stimulation for Data Classification

**DOI:** 10.3390/brainsci12091191

**Published:** 2022-09-03

**Authors:** Da Li, Molan Li, Zhili Huang

**Affiliations:** 1 School of Mathematics and Information Science, Guangxi University, Nanning 530004, China; 2 School of Intelligent Manufacturing Engineering, Guangxi Electrical Polytechnic Institute, Nanning 530007, China

**Keywords:** neural model, inter-field interaction, interactive stimulation, supervised learning, data classification

## Abstract

Some neural models achieve outstanding results in image recognition, semantic segmentation and natural language processing. However, their classification performance on structured and small-scale datasets that do not involve feature extraction is worse than that of traditional algorithms, although they require more time to train. In this paper, we propose a brain-like neural model with interactive stimulation (NMIS) that focuses on data classification. It consists of a primary neural field and a senior neural field that play different cognitive roles. The former is used to correspond to real instances in the feature space, and the latter stores the category pattern. Neurons in the primary field exchange information through interactive stimulation and their activation is transmitted to the senior field via inter-field interaction, simulating the mechanisms of neuronal interaction and synaptic plasticity, respectively. The proposed NMIS is biologically plausible and does not involve complex optimization processes. Therefore, it exhibits better learning ability on small-scale and structured datasets than traditional BP neural networks. For large-scale data classification, a nearest neighbor NMIS (NN_NMIS), an optimized version of NMIS, is proposed to improve computational efficiency. Numerical experiments performed on some UCI datasets show that the proposed NMIS and NN_NMIS are significantly superior to some classification algorithms that are widely used in machine learning.

## 1. Introduction

Cognition is usually considered as the internal processes involved in environmental sensing and decision-making [1]. Classification is considered to be one of its main activities. In machine learning, image recognition, semantic segmentation, natural language processing and emotion analysis are ultimately categorized as classification problems [2,3,4]. There have been many algorithms proposed that seek to solve specific learning tasks by simulating the neural mechanisms of the brain’s cognitive process. They are called neural networks [5,6,7,8]. In 1958, Rosenblatt proposed the famous perceptron, which is regarded as the basic unit of modern neural networks, leading to the first boom in neural network research. The BP algorithm [9] was applied to neural models in 1986, which provided an error propagation method for feed-forward neural networks. A second boom in artificial neural network research followed.

In 1998, the convolution network LeNet [10] represented a breakthrough in the field of image classification. Then, a variety of deep neural networks were developed, such as VGG (Visual Geometry Group) [11], GoogleNet [12] and ResNet [13]. Deep neural models have achieved great successes [14,15,16,17] and some even surpass humans in the field of large-scale image recognition [18,19]. Google used a multi-head attention mechanism to improve the performance of a neural model in natural language processing [20,21], leading to a boom in the investigation of transformers [22,23,24,25]. The vision transformer (VIT) [26] applied a multi-head attention mechanism to computer vision and demonstrated excellent performance in image classification. Consequently, neural networks have become essential to the development of current machine learning.

Nevertheless, most neural models tend to be understood in terms of engineering functions, but ignore biological interpretability [27,28,29,30]. For example, what role does a neuron play in the whole network, and what rules are followed to transmit information between neurons? The lack of clarity regarding these mechanisms make it inevitable that there are uncertain risks, and that tedious manual parameter tuning is necessary when designing a neural network, which leads to demanding requirements of neural networks with regard to training conditions [31,32].

The success of neural networks depends on their excellent feature extraction capability resulting from their complex architectures and huge parameters, the optimizations of which are based on a BP algorithm that is a mathematical tool rather than a biological rule [33,34]. When the training set is small, or the learning task does not involve feature extraction, a BP algorithm is often not necessary. We note that the classification performance of neural networks is weaker than some traditional classification algorithms which are not based on optimization means for structured and small-scale datasets.

In this paper, we propose a brain-like neural model NMIS that is applied to the field of small-scale and structured data classification. It simulates hierarchical brain structures and their neural activities using two neural fields: a primary field and a senior field. Its primary neurons (PN) are considered to be the representation of real instances in the feature space and its senior neurons (SN) represent category patterns.

At present, the research findings on memory generation generally agree that the connection between two neurons is usually built according to synaptic plasticity rules, such as the Hebbian rule and spike-timing-dependent plasticity (STDP). In NMIS, when a PN that an instance corresponds to, and an SN that stores this instance’s category pattern, are activated by the external input stimulation simultaneously, they tend to establish an inter-field connection, which represents the formation of memory [35,36,37]. We consider that the inter-field connections between PNs and SNs also follow the Hebbian rule.

In addition, in the NMIS’s primary field, the PNs corresponding to instances with the same category pattern form a subpopulation [38,39,40]. Once a PN is activated, it tends to trigger others in the same subpopulation and to inhibit unrelated neurons. The neuroscience literature reports that when a neuron discharges in the brain, it will release a chemical substance at the end of the axon which can be transmitted to connected neurons through the synaptic gap to activate or inhibit them [41,42]. NMIS’s PNs employ a similar information transmission mechanism to activate or inhibit other PNs in the primary field, called interactive stimulation. Inter-field interaction is defined as the stimulation of PN to SN, which is unidirectional and based on inter-field connections. Through it, an SN can perceive the PNs in an excited state.

The complete cognitive process of NMIS is described as follows: (1) External input stimulation activates a new PN; (2) Through interactive stimulation, other PNs in the same subpopulation as the activated PN are activated; (3) Through inter-field interaction, an SN is activated by these excited PNs, causing a category pattern to be perceived.

Consequently, in NMIS, the roles played by all neurons are clearly defined and the information interaction mechanism among the neurons is determined explicitly by the interactive stimulation (DOG function) or Hebbian rule instead of a BP strategy. So, compared with traditional neural networks, NMIS does not contain complex optimization steps or involve manual intervention in parameter selection, avoids the “black box” issue, and exhibits good performance in small-scale data classification.

Finally, we analyze the difficulties faced by the proposed NMIS in processing for large-scale data classification. Based on NMIS, involving a cooperation with the nearest neighbor strategy, we propose the NN_NMIS, an optimized version of NMIS, that focuses on large instance learning. The main contributions of the paper are summarized as follows:

- We propose a brain-like NMIS that consists of the primary field and the senior field, simulating hierarchical brain structures and their neural activities. The connections between neurons in NMIS are determined explicitly by the interactive stimulation or Hebbian rule instead of a BP strategy. So, the NMIS model does not require a complex optimization process.
- We propose a supervised learning algorithm based on the NMIS model. This algorithm applies a clear neural mechanism that is similar to real cognitive processes and avoids the “black-box” problem. Numerical results confirm that the proposed methods perform better than some widely used classification algorithms.
- We propose NN_NMIS for structured and large-scale data classification by combining the NMIS with a nearest neighbor strategy. The experimental results demonstrate that its performance is better than that of traditional classification algorithms.

## 2. Materials and Methods

### 2.1. The NMIS Model

There are two neural fields in NMIS to simulate the neural behaviors of cognition: the primary field and the senior field. The neurons in the primary field correspond one-to-one to the real instances in the feature space and the neurons in the senior field correspond one-to-one to the category pattern. Similar hierarchical structures are also commonly discussed in brain function research [43,44,45].

Recent investigations in cognitive science and neuroscience confirm that some neurons in the primary visual cortex V1 exhibit sharp selectivity for motion direction, and some of them possess the same preference and respond similarly to stimulation [40,46,47,48,49,50]. In NMIS, these neurons with similar behavior are defined as the PNs whose corresponding instances belong to the same category pattern. They form a subpopulation in NMIS’s primary neural field and are easily activated by each other. The information interaction among PNs is achieved through interactive stimulation, the strength of which is determined by an interaction kernel. Once a PN is activated, its interactive stimulation can activate other neurons that have a similar preference to it and inhibit unrelated neurons. When a PN that an instance corresponds to, and an SN that stores this instance’s category pattern, are activated by the external stimulation at the same time, they establish an inter-field connection. The inter-field interaction among the PNs and the SNs is based on the inter-field connections via which the excited PNs can activate the SNs that store their category pattern.

The interactive stimulation may be unidirectional for some PNs. In NMIS’s primary field, each PN has a resting activation. If a PN has not established the inter-field connection with an SN, it is called an implicit primary neuron (IPN). The resting activation of all IPNs is a uniform value and named the intrinsic resting activation [51,52,53]. The other PNs are called explicit primary neurons (EPN). The IPNs cannot receive interactive stimulation from other PNs, but the activated IPNs can exert interactive stimulation on EPNs. So, the IPNs can only be activated by the external input stimulation from their corresponding instances. Although the activation of neurons is generally positive, to prevent the PNs from being in an excitable state for a long time, we set their resting activation to be a negative value. In particular, the resting activation of the IPNs should be so small that they cannot be activated by the interactive stimulation from other PNs. Similar properties are also applied to SNs. We abbreviate the explicit senior neuron to ESN and the implicit senior neuron to ISN.

Based on these interaction mechanisms of NMIS, the activation of neurons in the two fields would be influenced by the external input stimulation, the interactive stimulation, the inter-field interaction and their resting activation. The cognitive ability of the model ultimately depends on the responses of the ESNs to external input stimulation.

**Figure 1:**
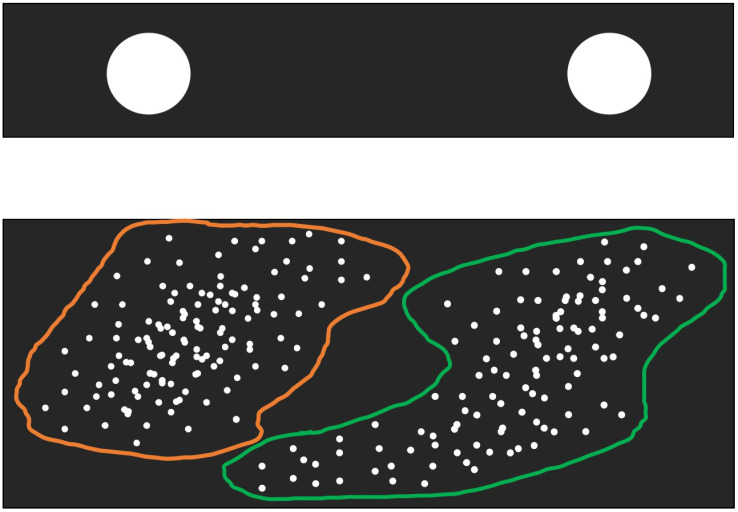
The primary neural field (below) and senior neural field (above) of NMIS. There are two subpopulations in the primary field, which correspond to two SNs. The white ones are IPNs (in the primary field) and ISNs (in the senior field). All of them are implicit until the inter-field connections are established.

We consider the binary classification problem as an example to illustrate the two neural fields of NMIS in Figure 1. We introduce the details of NMIS’s interaction mechanism in the following discussion. The symbols used in this paper are shown in Table 1.

#### 2.1.1. The Primary Neural Field

The primary neural field is used to correspond to the feature space of the real instances. Each primary neuron corresponds to a real instance. Suppose there are *m* PNs (IPNs and EPNs). Since the activation of PNs is mainly affected by interactive stimulation, external input stimulation and resting activation, we define the activation of the *i*th PN as $f_{i}\left(t\right)$, i=1,2,⋯,m which is a time-continuous dynamic form. If it is an EPN, $f_{i}\left(t\right)$, i=1,2,⋯,m satisfies the Equation:

(1) $$\begin{array}{ccc} \tau \dot{f_{i}}\left(t\right) & = & C_{f}\eta (\sum_{k=1}^{m}\omega \left(z_{i}-z_{k}\right)\phi (f_{k}\left(t\right)))\\ & + & h_{f,i}+e_{f,i}\left(t\right)+f_{i}\left(t\right) \end{array}$$

τ decides the evolution rate of $f_{i}$. For simplicity, we usually let τ=1. $e_{f,i}\left(t\right)$ be an external input stimulation from the *i*th EPN corresponding instance. The strength of it is usually supposed to be 1 when there is external stimulation input and 0 when there is not external stimulation input.

The term

$$C_{f}\eta \left(\sum_{k=1}^{m}\omega \left(z_{i}-z_{k}\right)\phi \left(f_{k}\left(t\right)\right)\right)$$

describes the interactive stimulation received by the *i*th EPN. The function $\sum_{k=1}^{m}\omega \left(z_{i}-z_{k}\right)$ is a DOG (difference of Gaussian) function that determines the interactive stimulation strength. Its form is

(2) $$\begin{array}{c} \omega \left(z_{i}-z_{k}\right)=A\exp\left(-\frac{d(z_{i},z_{k})}{2\sigma_{1}^{2}}\right)-B\exp\left(-\frac{d(z_{i},z_{k})}{2\sigma_{2}^{2}}\right), \end{array}$$

where $\sigma_{1}$ and $\sigma_{2}$ are two positive constants, describing the excitatory and inhibitory interactive stimulation scales of the PNs, respectively. Notice that $\sigma_{1}$ and $\sigma_{2}$ are two uniform values for all PNS (EPNs and IPNs). Generally, the inhibitory scale is about the triple of the excitatory scale, so, we let $\sigma_{2}=3\sigma_{1}$. The two exponential terms of $\omega (z_{i}-z_{k})$ are usually selected as the density functions of a normal distribution. So,

$$A=1/\sqrt{2\pi \sigma_{1}},B=1/\sqrt{2\pi \sigma_{2}}.$$

For convenience, let A−B=1 so that the maximum of $\omega (z_{i}-z_{k})$ is 1. Then we can obtain two definite values:

A=3/2,B=1/2.

Therefore, the $\omega (z_{i}-z_{k})$ is finally given as:

(3) $$\begin{array}{c} \omega \left(z_{i}-z_{k}\right)=\frac{3}{2}\exp\left(-\frac{d(z_{i},z_{k})}{2\sigma_{1}^{2}}\right)-\frac{1}{2}\exp\left(-\frac{d(z_{i},z_{k})}{2\sigma_{2}^{2}}\right). \end{array}$$

$d(z_{i},z_{k})$ is a distance function that can use the Euclidean distance, cosine distance, etc. The cosine distance is considered to be more suitable for processing image features extracted by deep networks.

ϕ(x) is an activation function for x∈R. It is monotonically increasing, non-negative and bounded. η(x) is a monotonically increasing threshold function. It describes the response of the *i*th EPN to the interactive stimulation that it receives, satisfying

$$\lim_{x\to +\infty }\eta \left(x\right)=1,$$

$$\lim_{x\to -\infty }\eta \left(x\right)=-1.$$

The functions ϕ(x) and η(x) are given as:

(4) $$\begin{array}{c} \phi \left(x\right)=\left\{\begin{array}{cc} 1-\exp(-x), & x>0\\ 0, & x\leq 0 \end{array}\right., \end{array}$$

and

(5) $$\begin{array}{c} \eta \left(x\right)=\left\{\begin{array}{cc} 1-\exp(-x), & x>0\\ -1+\exp(x), & x\leq 0 \end{array}\right.. \end{array}$$

$C_{f}$ is a positive constant that is employed to limit the interactive stimulation. In most cases, $C_{f}=1.$

We define $h_{f}$ as the resting activation of all EPNs, $h_{f,i}=h_{f}$. For EPN, we specify that it can be activated by strong interaction stimulation from other PNs (EPNs and IPNs) even without any external input stimulation. So, let

(6) $$\begin{array}{c} h_{f,i}=\alpha \cdot -\left(max\left(C_{f}\eta \left(\sum_{k=1}^{m}\omega \left(z_{i}-z_{k}\right)\phi \left(f_{k}\left(t\right)\right)\right)\right)\right), \end{array}$$

where α is a positive constant. Normally, take α=0.2, so, $h_{f,i}=h_{f}=-0.2.$

Provided that the *i*th PN is an IPN, its activation behavior $f_{i}\left(t\right)$, i=1,2,⋯,m is given as the following Equation:

(7) $$\begin{array}{ccc} \tau \dot{f_{i}}\left(t\right) & = & C_{f}\eta (\sum_{k=1}^{m}\omega \left(z_{i}-z_{k}\right)\phi (f_{k}\left(t\right)))\\ & + & H_{f,i}+e_{f,i}\left(t\right)+f_{i}\left(t\right) \end{array}$$

$H_{f,i}$ is the intrinsic resting activation of the *i*th IPN. It is assumed that the IPN can only be activated by external input stimulation. Therefore,

(8) $$\begin{array}{c} H_{f,i}\leq -\left(max\left(C_{f}\eta \left(\sum_{k=1}^{m}\omega \left(z_{i}-z_{k}\right)\phi \left(f_{k}\left(t\right)\right)\right)\right)\right). \end{array}$$

Generally, let $H_{f,i}=H_{f}=-1.$

Further, in order to improve computational efficiency, the interaction term of IPNs is canceled and the external input stimulation is changed to strong stimulation. The updated activation behavior of the *i*th IPN is satisfied by the following Equation:

(9) $$\begin{array}{c} f_{i}\left(t\right)=H_{f,i}+2e_{f,i}\left(t\right) \end{array}$$

#### 2.1.2. The Senior Neural Field

In NMIS, the senior neural field is used to store the category patterns. Each SN corresponds to a category pattern. When an SN is activated, this indicates that a category pattern is perceived. For convenience, the activation behavior of all SNs, whether ISNs or ESNs, is not time-continuous and there is no interactive stimulation among SNs. The activation of the SNs depends on the inter-field interaction from the primary field and the external input stimulation. In addition, we stipulate that, when the activation of EPNs in the primary field is stable, the information can be transmitted to the senior neural field through the inter-field interaction.

Suppose that there are $m_{c}$ SNs (ISNs and ESNs). The activation of the *j*th SN (if it is an ESN) is described by $g_{j}$, $j=1,2,\cdots ,m_{c}$. It satisfies

(10) $$\begin{array}{ccc} g_{j} & = & C_{g}\phi \left(\frac{1}{C_{j}}\sum_{i=1}^{m}x_{j,i}\phi \left(f_{i}^{*}\right)\right)+h_{g,j}+e_{g,j}. \end{array}$$

$e_{g,j}$ is an external input stimulation from the *j*th ESN corresponding category pattern. It is 0 or 1.

$f_{i}^{*}$ represents the activation of the *i*th EPN when the activation of all the EPNs in the primary neural field is stable.

$x_{i,j}$ is the inter-field connection weight between the *i*th EPN and the *j*th ESN, which relies on the Hebbian rule and describes the inter-field interaction. Because ϕ(∗) is a non-negative threshold function, only the positive inter-field interaction is considered.

$C_{j}$ is a positive normalization constant to improve the robustness of the model, which is given as the number of the EPNs that connect to the *j*th ESN by the Hebbian rule. $C_{g}$ is a positive constant that controls the inter-field interaction from the primary field. Commonly, $C_{g}=1$

$h_{g}$ is defined as the resting activation of all ESNs. $h_{g,j}=h_{g}$. Similar to the discussion on the resting activation of EPNs, let

(11) $$\begin{array}{c} h_{g,j}=\alpha \cdot -(max\left(C_{g}\phi \left(\frac{1}{C_{j}}\sum_{i=1}^{m}x_{j,i}\phi \left(f_{i}^{*}\right)\right)\right). \end{array}$$

Then, $h_{g,j}=h_{g}=-0.2$

Provided that the *j*th SN is an ISN, its activation behavior $g_{j}$, $j=1,2,\cdots ,m_{c}$ is given as the following Equation:

(12) $$\begin{array}{ccc} g_{j} & = & C_{g}\phi \left(\frac{1}{C_{j}}\sum_{i=1}^{m}x_{j,i}\phi \left(f_{i}^{*}\right)\right)+H_{g,j}+e_{g,j}. \end{array}$$

Since no EPNs establish the inter-field connections with the ISNs, the term

$$C_{g}\phi \left(\frac{1}{C_{j}}\sum_{i=1}^{m}x_{j,i}\phi \left(f_{i}^{*}\right)\right)$$

is ignored. Therefore, the activation behavior of the *j*th ISNs is described by the Equation:

(13) $$\begin{array}{cc} g_{j} & =H_{g,j}+e_{g,j}. \end{array}$$

$H_{g,j}$ is the intrinsic resting activation of the *j*th ISN. If $e_{g,j}=1$, define

(14) $$\begin{array}{c} H_{g,j}\geq -e_{g,j}. \end{array}$$

Generally, let $H_{g,j}=H_{g}=-0.9$

Equations (1) and (10) give the activation behavior and interaction mechanism of NMIS’s neurons. Compared with the traditional BP neural network, NMIS has a defined neural mechanism and avoids the “black box” problem. Therefore, it is biologically plausible.

### 2.2. The Cognitive Process of NMIS

In NMIS, each PN corresponds to a specific exterior input stimulation. Specifically, in the classification task, this external stimulation comes from an instance in the feature space. The SNs are used to store the category patterns corresponding to these PNs. When an SN is activated, it indicates that a stored category pattern is recalled. Similarly, when a PN is activated, it illustrates that a specific instance is expressed.

We introduce the cognitive process of NMIS in two main parts: the memory generation stage and the external stimulation recognition stage.

#### 2.2.1. The Memory Generation of NMIS

In NMIS, the inter-field interaction between the primary field and the senior field is realized through the inter-field connections whose weights are given as $X={\left(x_{i,j}\right)}_{l,m_{c}}$. For a training instance $z_{i},i=1,2,\cdots ,l$ of $T_{rain}=\left\{z_{1},z_{2},\cdots ,z_{l}\right\}$, if the IPN that it corresponds to and the ISN that its category pattern corresponds to are activated by the external input stimulation from it and its category pattern, respectively, at the same time, they tend to establish an inter-field connection according to the Hebbian rule. Commonly, we let their inter-field weight be 1. So, we obtain

(15) $$\begin{array}{c} x_{i,j}=\left\{\begin{array}{cc} 1, & e_{f,i}\left(t\right)=e_{g,j}=1\\ 0, & else \end{array}\right.. \end{array}$$

**Figure 2:**
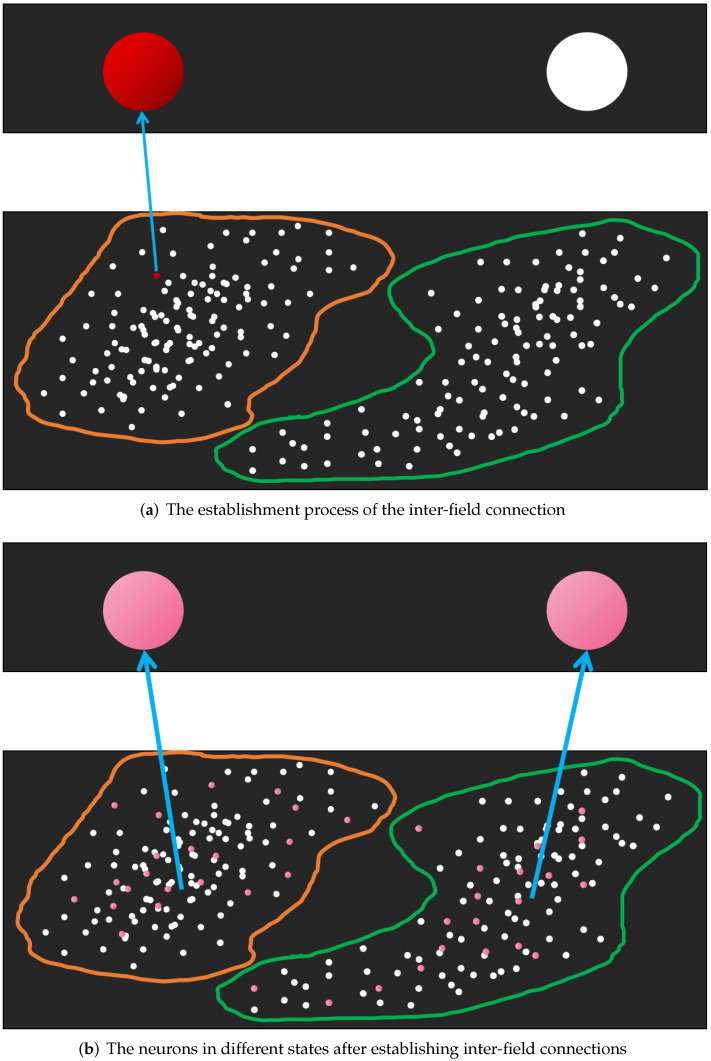
(**a**) shows the establishment process of the inter-field connection. (**b**) shows the neurons that have established inter-field connections. The white, pink and red ones represent implicit, explicit and activated neurons respectively. In (**a**), The thin blue arrows indicate an inter-field connection. The inter-field connections between the EPNs in the same subpopulation and ESN are indicated by a thick blue arrow in (**b**).

Figure 2 shows the memory generation process of NMIS, and the state that the NMIS is in after the memory is generated.

#### 2.2.2. The External Stimulation Recognition of NMIS

After the memory generation stage of the NMIS, the IPNs corresponding to the training instances are transformed into EPNs and can be easily activated again. If a new IPN is activated by an external stimulation from a test instance, some EPNs within its cognitive scale will be activated through its interactive stimulation and the unrelated EPNs will be inhibited. So, the cognitive scale of PN plays an important role in NMIS, which is determined by the interactive stimulation scale $\sigma_{1}$ and $\sigma_{2}$. Next, we offer a calculation method for the interactive stimulation scale using the distribution information of the instances.

Performing small-scale learning, we cannot determine the interactive stimulation scale by employing the distribution information of the training instances alone—the test instances must also be used. Let *D* be the l×m−l distance matrix that describes the distance between the training instances and test instances. Its elements   

$$d_{p,q}=d\left(z_{p},z_{q}\right)$$

where p=1,2,⋯,l and q=1,2,⋯,m−l. The d(∗) is a distance function. Let $d_{min,q},q=1,2,\cdots ,m-l$ be the minimum element in the corresponding column of *D* and $d_{max,q},q=1,2,\cdots ,m-l$ be the maximum element of each column. Then,

$$D_{min}=\left[d_{min,1},d_{min,2},\cdots ,d_{min,m-l}\right]$$

and

$$D_{max}=\left[d_{max,1},d_{max,2},\cdots ,d_{max,m-l}\right]$$

indicate the minimum and maximum distance among the training instances and test instances, respectively. We are unable to evaluate the range of the categories accurately, but it is reasonable to ascertain that the interactive stimulation induced by the new IPN activated by external input stimulation (from a test instance) should be large enough to activate at least one EPN. So, when handling the small-scale dataset, the interactive stimulation scale is given as:

$$\sigma_{1}=max\left(D_{min}\right).$$

Define

$$\sigma_{1,max}=max\left(D_{max}\right),$$

and

$$\sigma_{1,min}=min\left(D_{min}\right).$$

If the number of training instances for each category pattern is large. We can obtain enough internal information for the sub-populations only via the training instances’ distribution. Let $\hat{D}$ be an l×l matrix that describes the distance among the training instances. Its elements

$${\hat{d}}_{p,q}=d\left(z_{p},z_{q}\right),$$

where p=1,2,⋯,l and q=1,2,⋯,l. Let ${\hat{d}}_{max,q},q=1,2,\cdots ,l$ and ${\hat{d}}_{min,q},q=1,2,\cdots ,l$ be the maximum and minimum element in the corresponding column of $\hat{D}$, respectively. Then,

$${\hat{D}}_{max}=\left[{\hat{d}}_{max,1},{\hat{d}}_{max,2},\cdots ,{\hat{d}}_{max,l}\right],$$

and

$${\hat{D}}_{min}=\left[{\hat{d}}_{min,1},{\hat{d}}_{min,2},\cdots ,{\hat{d}}_{min,l_{j}}\right].$$

Because there is sufficient distribution information, it can be assumed that the interactive stimulation scale is

$$\sigma_{1}=mean\left({\hat{D}}_{max}\right).$$

Define

$$\sigma_{1,max}=max\left({\hat{D}}_{max}\right),$$

and

$$\sigma_{1,min}=min\left({\hat{D}}_{min}\right).$$

After the interactive stimulation scales are determined, we distinguish the category pattern of the test instances one-by-one. Notice that the external input stimulation to the ESNs is blocked, i.e., during the recognition process of NMIS, the ESNs only receive the inter-field interaction stimulation from the EPNs. So,

$$e_{g,j}=0,j=1,2,\cdots ,m_{c}.$$

For a test instance $z_{k},k=l+1,l+2,\cdots ,m$ of $T_{est}=\left\{z_{l+1},z_{l+2}\cdots ,z_{m}\right\}$, its initial state is the intrinsic resting activation. That is,   

$$f_{k}\left(t=0\right)=H_{f,k},k=l+1,l+2,\cdots ,m.$$

Let

e(f,k)(t)=1,k=l+1,l+2,⋯,m

to activate the the corresponding IPN.

Via the interactive stimulation, some EPNs that belong to the same subpopulation as this activated IPN are triggered and the unrelated EPNs are inhibited. When the primary field is stable, the activation information of the EPNs is transmitted to the senior field through the inter-field interaction, resulting in the change in the ESN’s activation.

**Figure 3:**
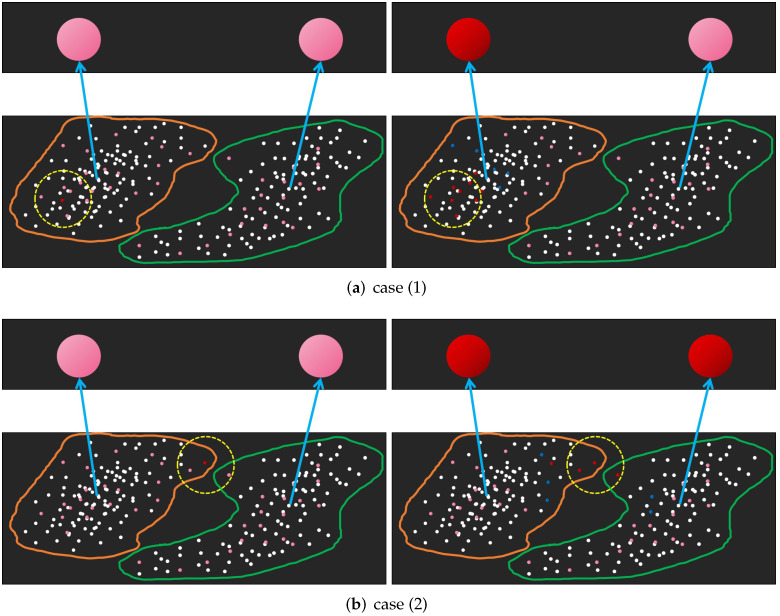

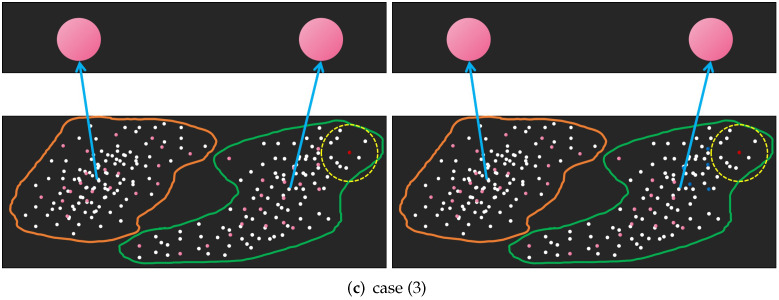
(**a**–**c**) show that only one ESN is activated, no more than one ESN is activated and no ESN is activated, respectively. The figures on the left describe the new IPN that is activated by external input stimulation and its excitatory interaction range (the yellow dotted line). The figures on the right describe the EPNs that are activated (red) or inhibited (blue) by interactive stimulation and the ESNs activated by inter-field interaction.

There are three situations that should be considered: (1) Only one ESN is activated; (2) No more than one ESN is activated; and (3) No ESN is activated. Figure 3 shows their general situation.

The first case is ideal because only one category pattern is perceived. So, the external input stimulation (test instance) is labeled as this perceived category pattern. For the second case, if the activation of an activated ESN is much higher than that of other ESNs, the external input stimulation is labeled accordingly. For the other cases, the interactive stimulation scale should be adjusted. Algorithm 1 demonstrates the details of our scale-adjusting algorithm in which the number of the activated ESNs is abbreviated as $A_{esn}$. The complete cognitive algorithm is shown in the Algorithm 2.
**Algorithm 1** Scale-Adjusting Algorithm.  1:INPUT   2:$A_{esn}$, $\sigma_{1}$,$\sigma_{1,max}$, $\sigma_{1,min}$, λ;  3:
  4:OUTPUT     5:$\sigma_{1}$, $\sigma_{1,max}$,$\sigma_{1,min}$;  6:
  7:**if**$A_{esn}=0$**then**  8:    **if**
$\sigma_{1,max}-\sigma_{1}<\epsilon$ **then**  9:        Let $\sigma_{1,max}=\sigma_{1,max}/\lambda$;10:    **end if**11:    Let $\sigma_{1,min}=\sigma_{1}$;12:    Calculate $\sigma_{1}=\sigma_{1}+\lambda \left(\sigma_{1,max}-\sigma_{1}\right)$;13:**else**14:    **if** $A_{esn}>1$ **then**15:        **if** $\sigma_{1}-\sigma_{1,min}<\epsilon$ **then**16:           Let $\sigma_{1,min}=\lambda \sigma_{1,min}$;17:        **end if**18:        Let $\sigma_{1,max}=\sigma_{1}$;19:        Calculate $\sigma_{1}=\sigma_{1}-\lambda \left(\sigma_{1}-\sigma_{1,min}\right)$;20:    **end if**21:**end if**

**Algorithm 2** Recognition Algorithm.  1:INPUT     2:$T_{est}=\left\{z_{l+1},z_{l+2}\cdots ,z_{m}\right\}$, $x_{i,j}$, i=1,2,⋯,l and $j=1,2\cdots ,m_{c}$;  3:
  4:OUTPUT     5:$\hat{Z_{test}}$;  6:
  7:Let $f_{i}\left(t=0\right)=-0.2$, $g_{j}=-0.2$, where i=1,2,⋯,l and $j=1,2\cdots ,m_{c}$;  8:**for** each $z_{k}\in T_{est}$ **do**  9:    Let $e_{f,k}\left(t\right)=1$;10:    Compute the stationary solution $f_{i}^{*}$, i=1,2,⋯,l of the Equation (1);11:    Compute the stationary solution $g_{j},j=1,2\cdots ,m_{c}$ of the Equation (10);12:    **while** $A_{esn}\neq 1$ **do**13:        Adjust the scale $\sigma_{1}$ by Algorithm 1;14:        Compute the stationary solution $g_{j},j=1,2\cdots ,m_{c}$ of the Equation (10);15:    **end while**16:    Let $\hat{z_{k}}=z^{\left(r\right)}$, where *r* is the category pattern that stored by the senior neuron with the highest activation;17:
**end for**
18:$\hat{Z_{test}}=\left\{\hat{z_{k}}\right\}$, k=l+1,l+2,⋯,m.

### 2.3. The NN_NMIS

The proposed NMIS has a vivid neural mechanism, which can effectively identify the external input stimulation—but it is not an efficient model. The number of its EPNs is equal to the number of the training instances. When the training set is large, a very complex interactive stimulation needs to be calculated, which means that NMIS faces unacceptable computational and storage requirements.

**Figure 4:**
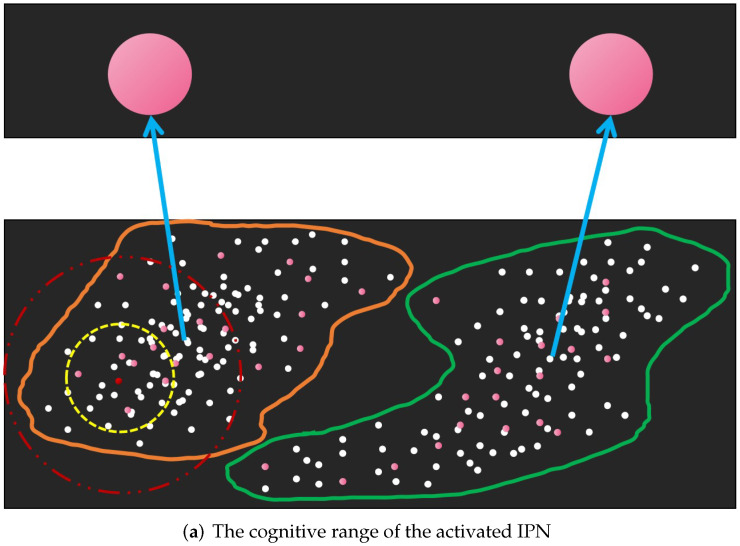

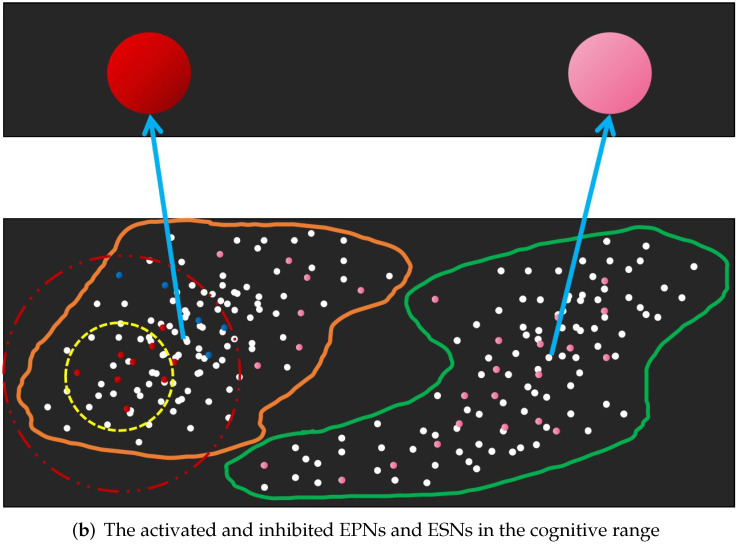
(**a**) shows the cognitive range of the activated IPN: the yellow dotted line is the excitatory interaction range and the red dotted line is the inhibition interaction range. (**b**) describes the activated and inhibited EPNs and ESNs in the cognitive range.

Not all of the interaction information of EPNs is meaningful. In Figure 4, the interaction stimulation induced by the activated IPN to the EPNs that are outside the red dotted line (cognitive scale) is so small that none of the EPNs can be activated or inhibited, but the model needs to spend most of the resources calculating them. This is inefficient and unreasonable.

In this section, we propose the nearest neighbor NMIS (NN_NMIS) which is mainly used to deal with large-scale data classification. Firstly, we select one representative EPN for each subpopulation, which is the weighted combination of all EPNs belonging to the same subpopulation. Then, the activated IPN only applies interactive stimulation to its nearest *K* EPNs and these representative EPNs.

The value of *K* is flexible. However, based on the premise that adequate meaningful EPNs are included, it should be as small as possible. According to the prior information, we can not get an optimal *K*, but we can reasonably assume that the number of these meaningful EPNs should not be more than that of the EPNs contained in the largest sub-population in the primary field. So, let

$$K<max\left(l_{j}\right),j=1,2,\cdots ,m_{c},$$

where $l_{j}$ is the number of EPNs in the *j*th sub-population. Generally, let

K=10

The representative EPNs should contain much information about the EPNs closing to the sub-population center and little information about the marginal EPNs. To generate high-quality representative EPNs, we propose a weight initialization method based on the interactive stimulation among the EPNs in the same sub-population.

Applying external input stimulation to activate all EPNs that belong to the same sub-population simultaneously, the EPNs corresponding to the sub-population center receive strong interactive stimulation from other EPNs and the interactive stimulation received by the EPNs corresponding to the sub-population boundary is tiny. Inspired by this idea, we initialize the weights using the interactive stimulation received by all EPNs as prior knowledge.

Suppose that there are $l_{j}$ EPNs in the *j*th sub-population. Denote $ITS_{j,k}$ as the interactive stimulation received by the *k* EPN of the *j*th sub-population. It is described by an instantaneous Equation:

$${ITS}_{j,i}=C_{f}\eta \left(\sum_{k=1}^{l_{j}}\omega \left(z_{j,i}-z_{j,k}\right)\phi \left(h_{f,j,k}+e_{f,j,k}\right)\right),$$

where $l_{j}$ is the number of the EPNs in the *j*th subpopulation and $e_{f,j,k}=1$. The representative EPN is abbreviated to REPN in the following equations. It is given by:

(16) $$\begin{array}{c} REPN_{j}=\sum_{k=1}^{l_{j}}w_{j,k}z_{j,k},j=1,2,\cdots ,m_{c}. \end{array}$$

where $m_{c}$ is the number of subpopulations. The weight vector $w_{j}={\left[w_{j,1},w_{j,2},\cdots ,w_{j,l_{j}}\right]}^{T}$ is given by the following equation:

(17) $$\begin{array}{c} w_{j}=Softmax\left(ITS_{j}\right), \end{array}$$

where $ITS_{j}={\left[ITS_{j,1},ITS_{j,2},\cdots ,ITS_{j,l_{j}}\right]}^{T}$. The function Softmax(∗) is a normalizing function that is used to ensure $\sum_{k=1}^{l_{j}}w_{j,k}=1$.

## 3. Results

In this section, to illustrate the classification performance of the proposed NMIS and NN_NMIS, we tested them on some real datasets. The details of the experimental datasets are described in Table 2, all of which were selected from the University of California Irvine (UCI) repositories (https://archive.ics.uci.edu/, accessed on 1 August 2022) and only underwent simple preprocessing, such as deleting the instances with null values. The original attribute values and dimensions of the datasets were not changed. The KNN and SVM were used for contrast, because they are considered two effective classification algorithms and are commonly used in various fields. We did not design comparative experiments with BP neural networks because their architectures are so diverse that the fairness of the experiments could not be guaranteed. To avoid accidental situations, all experimental results are the average of 30 rounds.

DERMATOLOGY, WINE, GERMAN, SEGMENTATION, PENDIGITS and SATELLITE datasets were selected to assess the model’s small-scale data classification capacities. The experiments were designed as one-shot learning and five-shot learning. There were one instance and five instances for each category pattern that were randomly selected as the training set; the rest were used as the test set. The comparison algorithms were: 1-NN (for one-shot learning), 3-NN (for five-shot learning) and SVM with a linear kernel (for all learning tasks).

**Figure 5:**
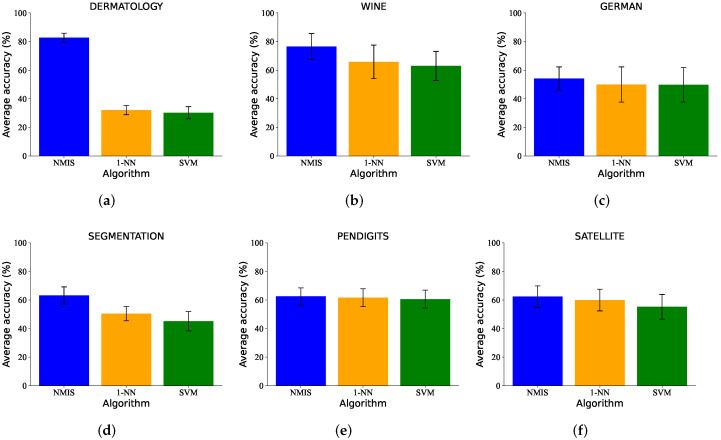
The one-shot learning results (%) of NMIS, KNN and SVM on (**a**) DERMATOLOGY, (**b**) WINE, (**c**) GERMAN, (**d**) SEGMENTATION, (**e**) PENDIGITS and (**f**) SATELLITE datasets.

Figure 5 shows the classification results of one-shot learning. We can see that the proposed NMIS outperformed the other algorithms in both accuracy and stability. On WINE, DERMATOLOGY and SEGMENTATION datasets, the accuracy of NMIS exceeded that of other algorithms by more than 15%.

**Figure 6:**
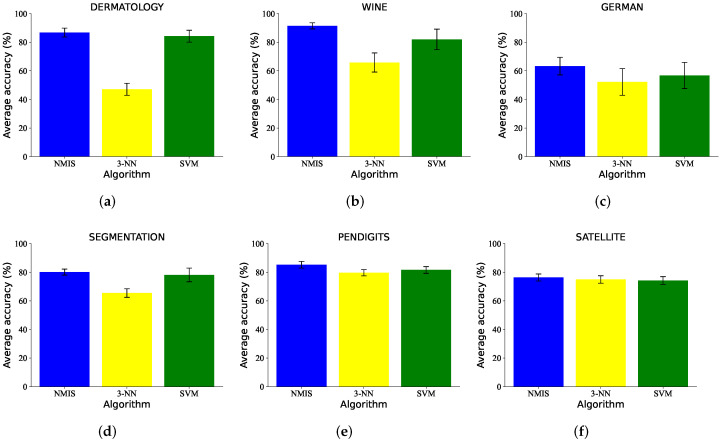
The five-shot learning results (%) of NMIS, KNN and SVM on (**a**) DERMATOLOGY, (**b**) WINE, (**c**) GERMAN, (**d**) SEGMENTATION, (**e**) PENDIGITS and (**f**) SATELLITE datasets.

**Figure 7:**
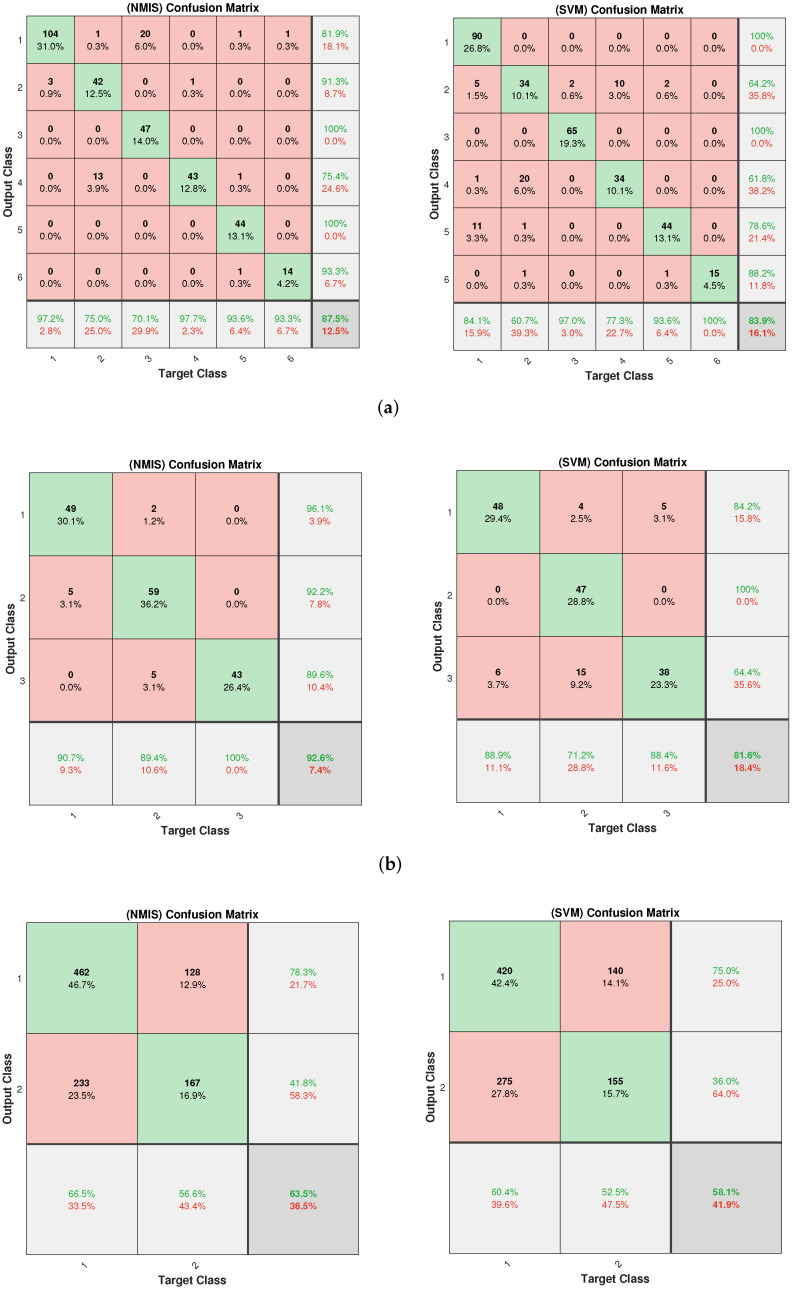
The confusion matrix of NMIS and SVM on (**a**) DERMATOLOGY, (**b**) WINE and (**c**) GERMAN datasets. Each orange square represents the number of wrongly predicted instances. The main diagonal represents the number of correctly predicted instances. The bottom and right light gray rectangles indicate the prediction accuracy of the corresponding instance categories.

**Figure 8:**
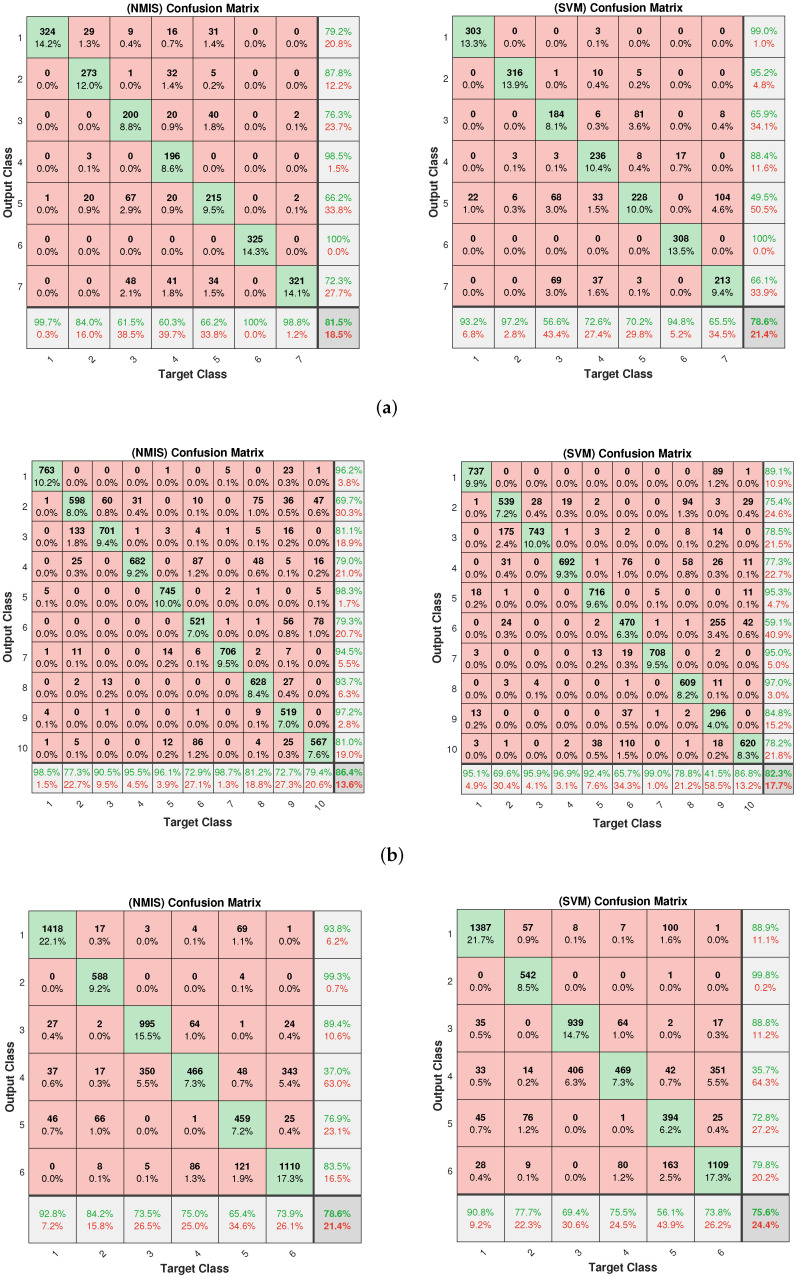
The confusion matrix of NMIS and SVM on (**a**) SEGMENTATION, (**b**) PENDIGITS and (**c**) SATELLITE datasets. Each orange square represents the number of wrongly predicted instances. The main diagonal represents the number of correctly predicted instances. The bottom and right light gray rectangles indicate the prediction accuracy of the corresponding instance categories.

The classification results of five-shot learning are shown in Figure 6. It can be seen that the accuracy and robustness of NMIS were still better than that of 3-NN. Significant performance gaps were obtained on the WINE, DERMATOLOGY, GERMAN and SEGMENTATION datasets. We observed that a similar accuracy to NMIS was obtained by SVM. Using the confusion matrix, we visualize the classification results of NMIS and SVM in Figure 7 and Figure 8. We can see that NMIS showed stable recognition ability for rare categories.

**Figure 9:**
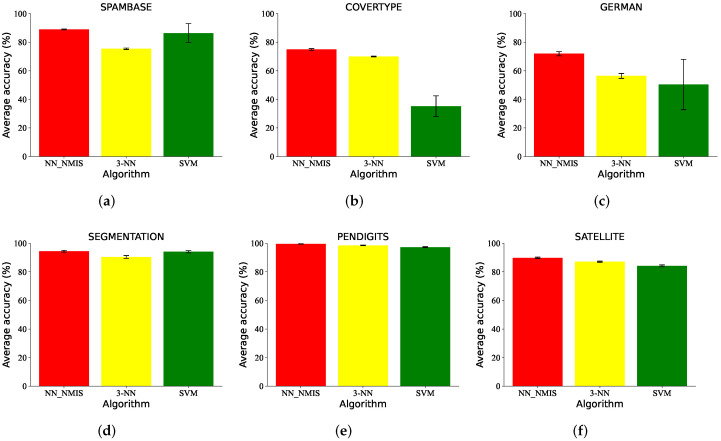
The large-scale data classification results (%) of NN_NMIS, KNN and SVM on (**a**) SPAMBASE, (**b**) COVERTYPE, (**c**) GERMAN, (**d**) SEGMENTATION, (**e**) PENDIGITS and (**f**) SATELLITE datasets.

To demonstrate the large-scale data classification capacity of NN_NMIS, we tested it on some large datasets: SPAMBASE, COVERTYPE, GERMAN, SEGMENTATION, PENDIGITS and SATELLITE. All datasets contained more than one thousand instances. A total of 30% of instances for each category pattern for SPAMBASE, GERMAN, SEGMENTATION, PENDIGITS and SATELLITE were randomly chosen as the training set and the rest were applied as the test set. The COVERTYPE dataset was so large that only 0.001% of instances for each category pattern were selected as the training set. The 3-NN and SVM were employed for comparison. For NN_NMIS, K=10 for all datasets. The results are shown in Figure 9. We can clearly see that the stability of the NN_NMIS was so good that there were no visible fluctuations. For the SPAMBASE, COVERTYPE and GERMAN datasets, the accuracy of the model was significantly better than for the other algorithms, especially SVM.

## 4. Discussion

Human beings show excellent cognitive ability, which depends on complex neuronal behavior mechanisms. Some scientists have sought to imitate these mechanisms to enable machines to acquire similar learning ability. The related research outcomes are called artificial neural networks (ANN). From the initial perceptron to the current diversified architectures (RNN, CNN, GNN and Transformer), the development of ANNs has undergone many reformulations, which have achieved extraordinary success in various fields [54,55]. However, the neural mechanism of ANNs is deficient and their training conditions are rigorously induced by the BP strategy, which leads to their poor biological rationale and unsatisfactory small-sample learning ability [27,28,29,30,33].

Some properties of real neurons can be applied to neural models to improve their biological interpretability. The BP algorithm is not necessary when dealing with structured datasets that do not involve data feature extraction. In this paper, we propose a brain-like neural model with interactive stimulation (NMIS) that focuses on structured and small-scale data classification. In contrast to traditional BP neural networks, the inspiration for NMIS originates from real cognitive processes. There are two neural fields in NMIS that are used to simulate the neural activation of primary and senior visual cortices, respectively. The information transmission and inter-field connections among the neurons in NMIS depend on the interactive stimulation and synaptic plasticity. Thus, its neural mechanism is clear. In addition, all parameters of the proposed model are reasonably selected according to cognitive science or independently designed according to the datasets. So, there are no complicated optimization steps and manual parameter adjustments in NMIS. To solve the unacceptable computing and storage requirements faced by NMIS when processing large-scale data classification, we propose NN_NMIS, an optimized version of NMIS, involving combining the nearest neighbor strategy, for large-scale data classification processing. Benefiting from a rational cognitive mechanism, NMIS and NN_NMIS show better classification abilities than other algorithms.

We do not doubt the excellent results achieved by neural networks. NMIS does not have feature extraction capability, which makes it unable to handle unstructured data directly. So, the massive structured data provided by neural networks is necessary. In the future, we intend to consider embedding NMIS into neural networks to achieve more interesting functions, such as emotion analysis, reinforcement learning and image recognition.

## 5. Conclusions

In this paper, we discuss some problems faced by traditional artificial neural networks, i.e., poor interpretability and demanding training conditions. Considering the excellent cognitive ability of human beings, some properties of neurons in the brain inspired us to design the NMIS model for small-scale and structured data classification. The neural mechanism of NMIS is clear. It consists of the primary field and the senior field, simulating the neural activation of primary and senior visual cortices, respectively. Its neurons transmit information through interactive stimulation and inter-field interaction, corresponding to the interaction and synaptic plasticity of real neurons. In contrast to the BP strategy, the memories of NMIS are stored as inter-field connections, which are based on the Hebbain rule and do not require strict optimization. Consequently, the proposed NMIS is biologically reasonable and efficient, and is essentially suitable for small-scale data classification. In addition, based on NMIS, we propose an NN_NMIS for large-scale learning, which only calculates the interaction information among a few important neurons. So, it is efficient. The numerical experiments on some UCI datasets demonstrate that the proposed NMIS and NN_NMIS are feasible and show better performance and generalization ability than some widely used classification algorithms in machine learning.

## Figures and Tables

**Table 1:** Symbols Table.

Symbols	Descriptions
$Z=\{z_{1},z_{2},\cdots ,z_{m}\}$	Set of all instances
$T_{rain}=\left\{z_{1},z_{2},\cdots ,z_{l}\right\}$	Set of all training instances
$T_{est}=\left\{z_{l+1},z_{l+2}\cdots ,z_{m}\right\}$	Set of all test instances
$\hat{Z}=\left\{{\hat{z}}_{1},{\hat{z}}_{2},\cdots ,{\hat{z}}_{m}\right\}$	Set of all instances’ category patterns
$\hat{Z_{train}}=\left\{{\hat{z}}_{1},{\hat{z}}_{2}\cdots ,{\hat{z}}_{l}\right\}$	Set of all training instances’ category patterns
$\hat{Z_{test}}=\left\{{\hat{z}}_{l+1},{\hat{z}}_{l+2}\cdots ,{\hat{z}}_{m}\right\}$	Set of all test instances’ labels
$N=\{{\hat{z}}^{\left(1\right)},{\hat{z}}^{\left(2\right)}\cdots ,{\hat{z}}^{\left(m_{c}\right)}\}$	Set of all category pattern names
*m*	The number of instances
$m_{c}$	The number of all category patterns
*l*	The number of training instances
$l_{j}$	The number of training instances with the category pattern ${\hat{z}}^{\left(j\right)}$

**Table 2:** Details of datasets.

	Instances	Classes	Attributes
**COVERTYPE**	581,012	7	54
**SPAMBASE**	4601	2	57
**GERMAN**	1000	2	20
**DERMATOLOGY**	366	6	34
**WINE**	178	3	3
**SEGMENTATION**	2310	7	19
**PENDIGITS**	7494	10	16
**SATELLITE**	6435	6	36

## Data Availability

The datasets generated during and/or analysed during the current study are available from the corresponding author on reasonable request.

## Abbreviations

The following abbreviations are used in this manuscript:

NMIS	The neural model with interactive stimulation
NN_NMIS	The nearest neighbor NMIS
PN	The primary neuron
SN	The senior neuron
IPN	The implicit PN
EPN	The explicit PN
ISN	The implicit SN
ESN	The explicit SN
